# Protective Effects of Unsaponifiable Matter from Perilla Seed Meal on UVB-induced Damages and the Underlying Mechanisms in Human Skin Fibroblasts

**DOI:** 10.3390/antiox8120644

**Published:** 2019-12-13

**Authors:** Hana Lee, Jeehye Sung, Younghwa Kim, Heon Sang Jeong, Junsoo Lee

**Affiliations:** 1 Division of Food and Animal Sciences, Chungbuk National University, Cheongju, Chungbuk 28644, Korea; dlgksk0514@naver.com (H.L.); hsjeong@chungbuk.ac.kr (H.S.J.); 2 Department of Food Science and Biotechnology, Andong National University, Andong, Gyeongbuk 36729, Korea; jeehye@anu.ac.kr; 3 School of Food Biotechnology & Nutrition, Kyungsung University, Busan 608-736, Korea; younghwakim@ks.ac.kr

**Keywords:** photoaging, skin, collagen, perilla seed meal, unsaponifiable matter

## Abstract

Unsaponifiable matter (USM) from perilla seed meal contains numerous phytochemicals, including tocopherols, phytosterols, squalene, and policosanols, that exhibit antioxidant and health-promoting properties. In this study, the protective effects of USM on UVB-induced skin aging were investigated in Hs68 cells. UVB irradiation decreased cell viability by 26% compared to the control. However, USM blocked UVB-induced cytotoxicity. Moreover, USM treatment significantly decreased the UVB-induced production of reactive oxygen species and attenuated the UVB-induced production and mRNA expression of matrix metalloproteinases (MMPs) by inhibiting the phosphorylation of mitogen-activated protein kinases and activator protein 1 (AP-1). Furthermore, UVB exposure led to a 49.4% reduction in collagen synthesis. However, USM treatment restored collagen synthesis through upregulation of the transforming growth factor beta (TGF-β)/Smad2/3 pathways. These data indicate that USM regulates the production of MMPs and collagen by modulation of the TGF-β/Smad pathway and AP-1 activity, suggesting that USM may be a useful anti-photoaging ingredient.

## 1. Introduction

Skin aging can be classified into extrinsic and intrinsic aging. Intrinsic aging is caused by a natural consequence of physiological changes over time [1], while extrinsic aging involves various factors, including pollutants, smoking, and ultraviolet (UV) light that accelerate the aging process [2,3]. Importantly, UV irradiation is the primary cause of extrinsic aging, and is characterized by severe hyperpigmentation, sagging, and wrinkling of the skin [4]. UV irradiation increases the intracellular generation of reactive oxygen species (ROS), considered the most important factor in the aging process, and up-regulates the phosphorylation level of mitogen-activated protein kinases (MAPKs). MAPKs regulate activator protein 1 (AP-1) [5]. When AP-1 is activated, it promotes the transcription of matrix metalloproteinases (MMPs), which in turn degrade the main components of extracellular matrix (ECM), including elastin, proteoglycans, and collagen [6]. Transforming growth factor beta (TGF-β) induces the synthesis and secretion of elastin and collagen [7] and down-regulates the expression of specific enzymes involved in collagen breakdown, including MMP1 and MMP3. Signaling by TGF-β is up-regulated by Smad2/3 and down-regulated by Smad7 [8,9]. Therefore, it is very important to down-regulate MAPK/AP-1 and Samd7 pathways and up-regulate TGF-β/Smad2/3 for maintaining skin health and recovering skin damage.

*Perilla frutescens* (L.) Britton, an edible plant belonging to the family Labiatae, is cultivated worldwide and used extensively in Asian countries [10,11]. Perilla seed meal (PSM) is generated as a by-product during the production of perilla seed oil and is an excellent source of unsaponifiable matter (USM), including tocopherols, policosanols, and phytosterols [12]. Tocopherols and tocotrienols are well known for their benefits for skin health. A recent study indicated that shea butter was a famous antioxidant and anti-inflammatory plant seed extract used in the cosmetic industry because of its high percentage of unsaponifiable compounds including tocopherols and phytosterols [13,14]. Rekik et al. also reported that the positive effect of vitamin E and phytosterols on collagen synthesis and skin wound healing is because these compounds prevent the damaging effects of free radicals and ensure the stability and integrity of biological membranes [15]. With increasing interest in the development of functional materials with very few side effects, studies are being conducted to find various plant extracts that inhibit skin aging. These materials represent various biological effects because they contain a large amount of physiological active substances. Despite containing abundant bioactive components, PSM is typically used as animal feed or natural fertilizer [16]. Previous studies have focused mainly on the purification and separation of proteins from PSM, and neither its protective effects nor its mechanisms of action have been reported to date [17]. In this study, therefore, we evaluated the protective effects of USM from PSM against UVB-induced photodamage in Hs68 cells. Additionally, we investigated the underlying mechanisms responsible for collagen degradation and synthesis, focusing on the MAPK/AP-1 and the TGF-β/Smad pathways.

## 2. Materials and Methods

### 2.1. Materials

2′7′-Dichlorofluorescein diacetate (DCFH-DA), dimethyl sulfoxide (DMSO), and 3-(4,5-dimethyl-thiazol-2-yl)-2,5-diphenyl-tetrazolium bromide (MTT) were purchased from Sigma-Aldrich (St. Louis, MO, USA). Antibodies against Smad7, TGF-β1, Smad2/3, p-Smad2/3, c-Jun, c-Fos, p-c-Jun, p-c-Fos, ERK1/2, p-ERK1/2, JNK, p-JNK, p38, p-p38, and β-actin were obtained from Santa Cruz Biotechnology (Santa Cruz, CA, USA) and Cell Signaling Technology (Danvers, MA, USA).

### 2.2. Preparation and Analysis of USM from Perilla Seed Meal

USM was prepared by saponification and the composition of USM was analyzed according to the method by Ham, Yoon, Kim, Kwak, Lee & Lee (2015) [18]. PSM (about 3 g) was weighed, and 15 mL of ethanol containing pyrogallol (6%, *w/v*) was added. After sonication for 2 min, 8 mL of potassium hydroxide in deionized water (60%, *w/v*) was added, and the mixture was flushed with nitrogen gas for 30 s. The contents were then saponified at 75 °C under reflux in a shaker water bath. After cooling, sodium chloride in water (2%, *w/v*) was added to adjust the ethanol concentration in the extraction medium. PSU was extracted with 30 mL of ethyl acetate/hexane (15:85, *v/v*) three times. The upper layer was collected, filtered, and evaporated under vacuum at 40 °C. The residues were dissolved in DMSO.

### 2.3. HPLC Analysis for Tocopherol and Tocotrienol

Analysis of tocopherols and tocotrienols was performed on a LiChrosphere^®^ Diol 100 column (250 × 4 mm i.d., 5 mm, Merck, Darmstadt, Germany) using a mobile phase of hexane/isopropanol (98.9:1.1, *v/v*) at a flow rate of 1.0 mL/min. The concentrations of tocopherols and tocotrienols in the samples were calculated using the average peak areas compared between standards and samples at 290 and 320 nm for excitation and emission wavelengths.

### 2.4. Gas Chromatography (GC) Analysis for Policosanol, Phytosterols and Squalene

The concentrations of policosanol, phytosterols and squalene were determined by GC using 5α-cholestane as an internal standard. The GC (Varian 3800; Varian Inc, Walnut Creek, CA, USA) was equipped with a SAC-5 fused-silica capillary column (30 m × 0.25 mm i.d.; Supelco, Bellefonte, PA, USA) and a flame ionization detector. The column was held at 280 °C for 1 min and programmed to rise to 300 °C at a rate of 2.0 °C/min. It was then held at 300 °C for 20 min. The carrier gas was helium, and the total gas flow rate was 20 mL/min. The injector and detector temperatures were 310 °C and 320 °C, respectively. A comparison of the retention times with those of standards permitted the identification of the policosanol, phytosterols, and squalene.

### 2.5. Cell Culture

The Hs68 cells (human dermal fibroblasts, obtained from ATCC CRL-1635, Manassas, VA, USA) were maintained in DMEM with 1% penicillin-streptomycin and 10% heat-inactivated FBS at 37 °C in 5% CO_2_ humidified air. The cells were seeded in 96-well plates (1 × 10^4^ cells /well) and were used between passage numbers 10 and 25.

### 2.6. USM Treatment and UVB Irradiation

To confirm the protective effect of USM, Hs68 cells were pretreated with various concentrations (1.0, 2.5 and 5.0 μg/mL) of USM in serum-free medium for 24 h. The culture medium was replaced with PBS, and the cells were, then, exposed to UVB irradiation (30 mJ/cm^2^) using GL20SE UVB lamps (Sankyo denki, Marine, Japan). The radiation intensity of UVB was monitored with a UV light meter (LT Lutron, UV-340A, Taipei, Taiwan). After exposure, the cells were immediately treated with USM in serum-free medium for additional 24 h. To examine the cytotoxicity of USM, cells were maintained under the same conditions without UVB irradiation. MTT assay was used to measure cell viability.

### 2.7. Measurement of Intracellular ROS

Intracellular ROS levels were quantified with a DCFH-DA fluorescent probe, as previously described [18]. Briefly, Hs68 cells were seeded in 96-well black plates at a density of 1 × 10^4^ cells /well, and pretreated with USM (1.0, 2.5, and 5.0 μg/mL) for 24 h, washed with PBS, and then irradiated with UVB (30 mJ/cm^2^). After 30 min, the cells were stained with 25 μM DCFH-DA and analyzed using a fluorescent spectrophotometer (LS-55, Perkin-Elmer, Norwalk, CT, USA).

### 2.8. Measurement of MMP1, MMP3, and Collagen

The production of MMP1 and MMP3 was measured using a commercially available ELISA kit (Merck &Co. Inc., Whitehouse Station, NJ, USA). Total collagen production was determined using the Sircol^TM^ kit (Biocolor, Carrickfergus, Northern Ireland).

### 2.9. Western Blot Analysis

The expression levels of proteins were confirmed as described elsewhere [19]. Proteins were separated and transferred to a nitrocellulose membrane (GE Healthcare, Buckinghamshire, UK). Equal amounts of proteins were separated on a 10% SDS-polyacrylamide gel and electrophoretically transferred to a nitrocellulose membrane (GE Healthcare, Buckinghamshire, UK). The membranes were blocked with tris-buffered saline/Tween 20 (TBST) containing 5% skim milk, and they were incubated for 12 h with primary antibodies. After washing with TBST, horseradish peroxidase-labeled secondary antibodies were added, and the blots incubated for 2 h. Protein bands were activated by chemiluminescence and visualized on an X-ray film.

### 2.10. Quantitative Real-Time PCR

To confirm the mRNA expression of the MMPs, the obtained cDNA was analyzed by qPCR (Applied Biosystems, Carlsbad, CA, USA) using the TaqMan Probe-Based Gene Expression analysis system in combination with the TaqMan Gene Expression Master Mix containing ROX (Applied Biosystems). Quantification of the *MMP1* (Hs00899658_m1), *MMP3* (Hs00968306_g1), and *GAPDH* (Hs02758991_g1) transcripts was performed using gene-specific primers.

### 2.11. Statistical Analysis

The results were represented as the mean ± standard error and all experiments were performed in triplicate. Statistical analysis was performed using GraphPad Prism software (GraphPad Software Inc., La Jolla, CA, USA) by Tukey’s post-hoc test.

## 3. Results and Discussion

### 3.1. Phytochemical Contents of USM

Numerous plant seeds are major sources of phytochemicals such as vitamins, flavonoids, and phenolic compounds. PSM is a by-product generated from oil extraction process [16]. USM contains high levels of policosanols, tocopherols, phytosterols, and squalene (Table 1). The extraction yield of USM was 3.4% (data not shown). The isomer of vitamin E, γ-tocopherol (T), was the most abundant component (330.67 mg/100 g USM), while tocotrienols (T3) were not detected. The major policosanol in USM was octacosanol (C28; 1802.98 mg/100 g of USM), followed by tetracosanol (C24; 857.72 mg/100 g of USM) and triacontanol (C30; 847.77 mg/100 g of USM). The main phytosterol was β-sitosterol (23016.25 mg/100 g of USM). The squalene content in USM was 1028.15 mg/100 g of USM. Argan oil, which contains tocopherols, polyphenols, squalene, triterpene alcohols, and sterols, has been used in skin care products and the treatment of skin infections [20]. A previous study further showed the protective effects of vitamin E on keratinocyte damage in a cell culture experiment [21], while Harrabi et al. [22] reported that the policosanol level in seed oils may contribute to their antioxidant. Phytosterols have also shown positive effects on skin barrier recovery [23]. These data indicate that these compounds, present in USM, may promote skin protection.

### 3.2. Effects of USM on Cell Viability, Protective Activity, and Total Collagen Production

**Figure 1:**
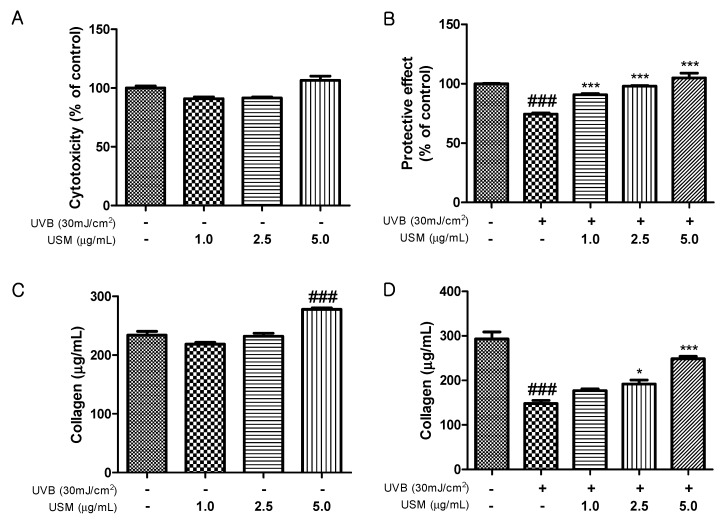
Effects of unsaponifiable matter (USM) from perilla seed meal on cytotoxicity (**A**), protective activity (**B**), total collagen production without UVB irradiation (**C**), and total collagen production with UVB irradiation (**D**) in Hs68 cells. UVB, ultraviolet b. Each value is expressed as the mean ± standard error (*n* = 3). Significance was tested using Tukey’s test. ^###^ *p* < 0.001 versus the non-irradiated control. * *p* < 0.05 and *** *p* < 0.001 versus the UVB-irradiated control.

UVB irradiation stimulates collagenase activity and increases wrinkle formation through the breakdown of collagen in the dermal [24,25]. We investigated the protective effects of USM against UVB-induced damage in Hs68 cells. The viability of Hs68 cells was not significantly altered by 48 h of incubation with USM up to 5 µg/mL (Figure 1A). Exposure to UVB reduced cell viability by 26% compared to control cells. However, USM treatment decreased this UVB-induced cytotoxicity (Figure 1B). We also assessed total collagen production in response to USM treatment and found that, at 5.0 µg/mL, USM increased total collagen production by 18.9% compared to that in control cells (Figure 1C). Although UVB irradiation decreased total collagen production (Figure 1D), USM treatment significantly mitigated UVB-induced collagen degradation.

### 3.3. Effects of USM Treatment on ROS Production

**Figure 2:**
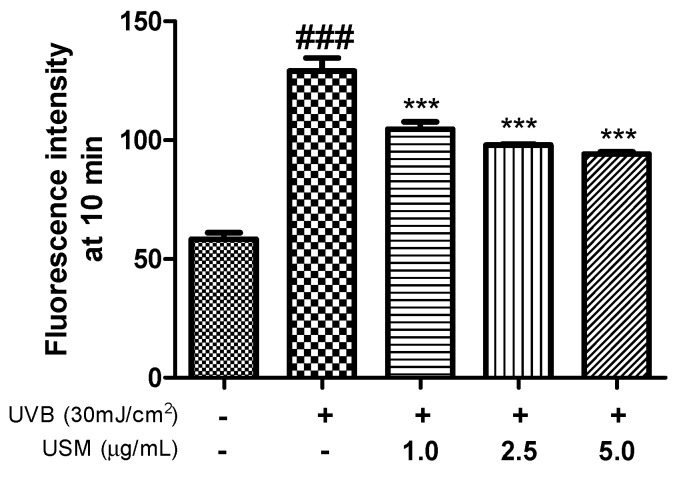
Effects of unsaponifiable matter (USM) from perilla seed meal on the production of reactive oxygen species (ROS) in Hs68 cells. UVB, Ultraviolet B. Each value is expressed as the mean ± standard error (*n* = 3). Significance was tested using Tukey’s test. ^###^ *p* < 0.001 versus the non-irradiated control. *** *p* < 0.001 versus the UVB-irradiated control.

Exposing cells to UVB irradiation can induce ROS production. ROS are known to play a key role in photoaging, triggering complex signaling pathways that result in the overexpression of MMPs or degradation of the ECM in connective tissues [26,27]. A previous study showed that macelignan, which is a natural lignan isolated from nutmeg, significantly inhibits UVB-induced increases in MMP1 expression by suppressing ROS production [28]. Oxidative stress both stimulates collagen degradation and inhibits the production of collagen [29]. Vitamin E, policosanol, phytosterol, and squalene are known to exhibit antioxidant activities and inhibit cholesterol oxidation [30]. Noh et al. [31] showed that pretreatment with a perilla seed meal extract significantly decreased ROS generation and protected HepG2 cells from oxidative stress induced by *t*-BHP. To understand whether USM inhibits UVB-induced MMP production and expression through antioxidative activities, intracellular ROS levels were determined after USM treatment. We found that USM treatment significantly reduced the UVB-induced ROS generation by 19.0, 24.1, and 27.1% at the concentration of 1.0, 2.5, and 5.0 µg/mL, respectively, compared to UVB irradiation (30 mJ/cm^2^) alone (Figure 2). These results indicate that USM inhibits ROS generation, which consequently elicits a protective effect.

### 3.4. Effects of USM Treatment on UVB-Induced MMP1 and MMP3 Secretion and mRNA Expression

**Figure 3:**
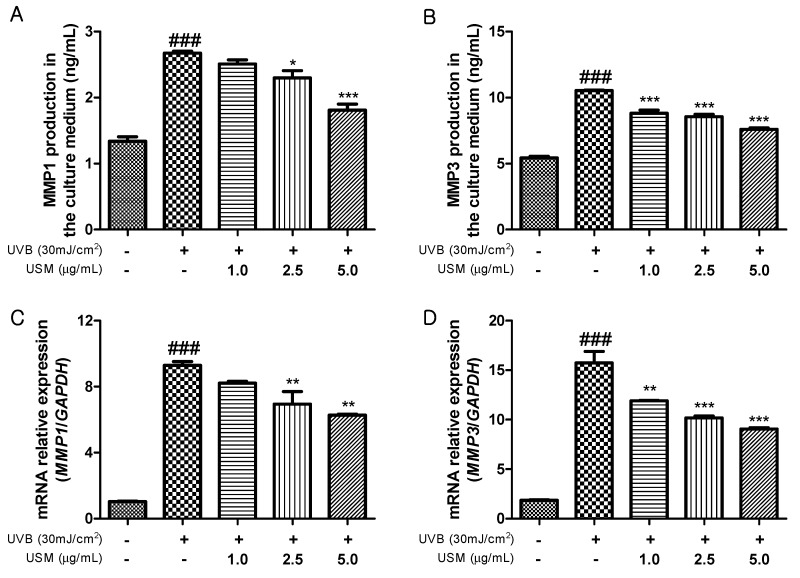
Effects of unsaponifiable matter (USM) from perilla seed meal on MMP1 and MMP3 production (**A,B**) and mRNA expression levels (**C,D**) in Hs68 cells. mRNA expression was examined by RT-qPCR. UVB, Ultraviolet B; MMP, matrix metalloproteinase. Each value is expressed as the mean ± standard error (*n* = 3). Significance was tested using Tukey’s test. ^###^ *p* < 0.001 versus the non-irradiated control. * *p* < 0.05, ** *p* < 0.01, and *** *p* < 0.001 versus the UVB-irradiated control.

MMPs play a crucial role in the mechanism of skin photoaging induced by UVB exposure [32]. UVB irradiation activates MMP1 that breaks down collagen [33]. MMP3 activates other MMPs such as MMP1, MMP7, and MMP9. Therefore, an MMP inhibitor may be effective at preventing UVB-induced skin sagging and wrinkling. We investigated the effect of USM treatment on MMPs in Hs68 cells. UVB irradiation significantly increased the production of MMPs compared to non-irradiated Hs68 cells (Figure 3A,B). However, USM treatment markedly decreased MMP1 and MMP3 production in a dose-dependent manner. The results also showed that MMP mRNA levels were also reduced in response to USM treatment (Figure 3C,D). Together, our results suggest that USM exerts an important role in protecting the skin from photoaging.

### 3.5. Inhibitory Effect of USM on AP-1 Activation

**Figure 4:**
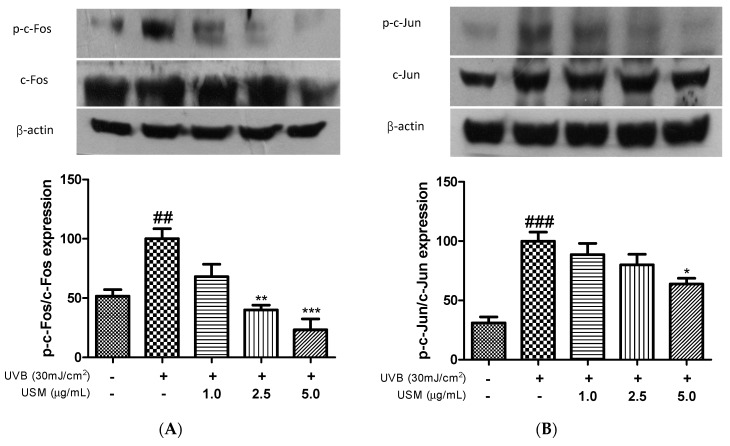
Effects of unsaponifiable matter (USM) from perilla seed meal on the phosphorylation of c-Fos (**A**) and c-Jun (**B**) in Hs68 cells. Protein expression was detected by Western blot using protein-specific antibodies. UVB, ultraviolet b. Each value is expressed as the mean ± standard error (*n* = 3). Significance was tested using Tukey’s test. ^##^ *p* < 0.01 and ^###^ *p* < 0.001 versus the non-irradiated control. * *p* < 0.05, ** *p* < 0.01 and *** *p* < 0.001 versus the UVB-irradiated control.

The AP-1 transcription factor, which is involved in MMP expression, is reportedly regulated by MAPKs [34]. A previous study showed that UVB irradiation increased the mRNA expression levels of *c-Jun* and *c-Fos* [32]. Park et al. reported that a *Eucalyptus globulus* extract markedly decreased the levels of UVB-induced phosphorylated c-Jun and c-Fos [35]. We confirmed that exposure to UVB irradiation led to the activation of AP-1. However, USM treatment suppressed c-Fos phosphorylation by 31.9, 59.9, and 76.7% at the concentration of 1.0, 2.5, and 5.0 µg/mL, respectively, compared to UVB irradiation alone (Figure 4A). We also assessed the expression level of c-Jun. Treatment with USM dose-dependently inhibited c-Jun phosphorylation by 11.4%, 20.0%, and 36.2%, respectively (Figure 4B). These results indicate that USM can directly suppress collagen degradation in human skin fibroblasts.

### 3.6. Inhibitory Effect of USM Treatment on MAPK Activation

**Figure 5:**
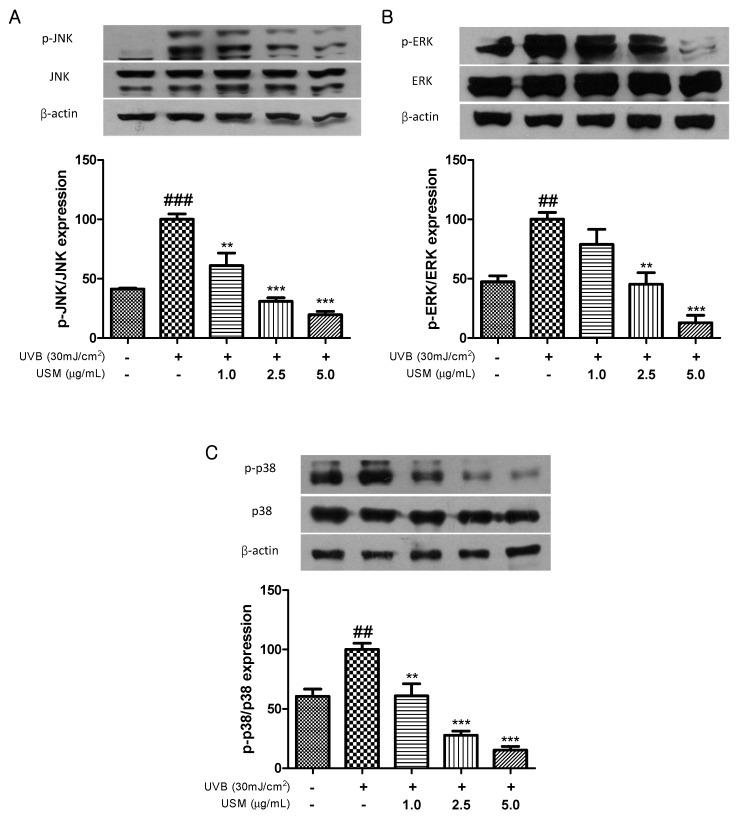
Effects of unsaponifiable matter (USM) from perilla seed meal on the phosphorylation of JNK (**A**), ERK (**B**), and p-38 MAP kinase (**C**) in Hs68 cells. Protein expression was detected by western blot using protein-specific antibodies. UVB, Ultraviolet B. Each value is expressed as the mean ± standard error (*n* = 3). Significance was tested using Tukey’s test. ^##^ *p* < 0.01 and ^###^ *p* < 0.001 versus the non-irradiated control. ** *p* < 0.01 and *** *p* < 0.001 versus the UVB-irradiated control.

MAPKs are involved in regulating the expression and production of MMPs. Rittie and Fisher [36] reported that oxidative stress resulting from ROS accumulation activates MAPK signaling through MAPK phosphorylation. Recent studies have shown that UVB-induced collagen breakdown is significantly ameliorated by inhibition of MAPK signaling [37,38]. As shown in Figure 5, UVB irradiation promoted MAPK phosphorylation, including that of p-JNK, p-ERK, and p-p38 MAP kinase. In contrast, USM treatment significantly inhibited the UVB-induced activation of JNK, ERK, and p38. Treatment with 5 µg/mL USM decreased the levels of p-JNK, p-ERK, and p-p38 by 80.3, 87.2, and 84.7%, respectively.

### 3.7. Regulatory Effect of USM on TGF-β1/Smad Pathway

**Figure 6:**
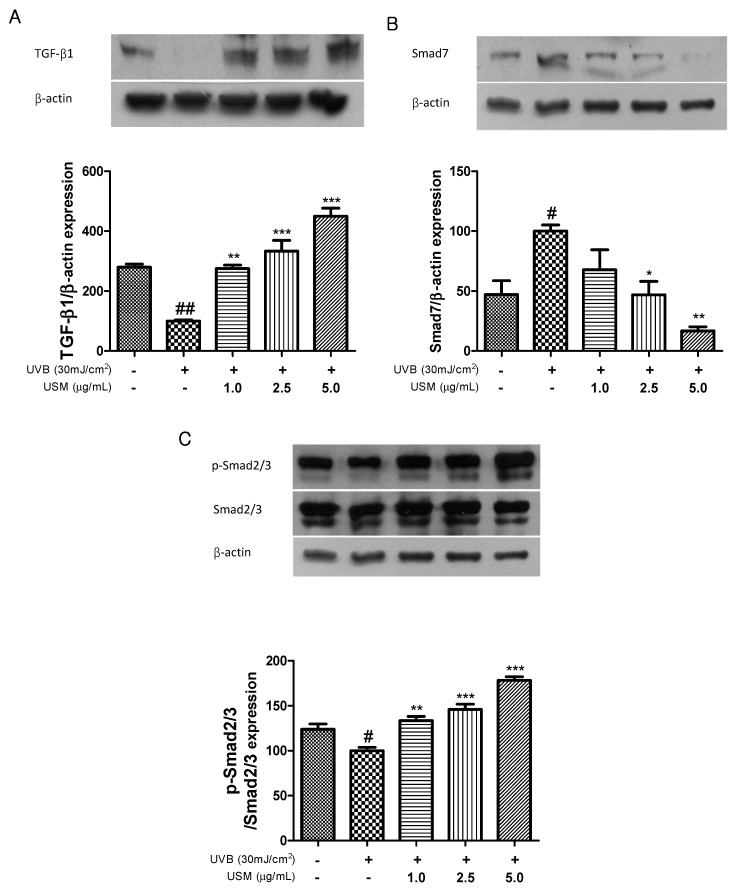
Effects of unsaponifiable matter (USM) from perilla seed meal on the expression of TGF-β1 (**A**), Smad 7 (**B**), and p-Smad 2/3 (**C**) in Hs68 cells. Protein expression was detected by western blot using protein-specific antibodies. UVB, Ultraviolet B. Each value is expressed as the mean ± standard error (*n* = 3). Significance was tested using Tukey’s test. ^#^ *p* < 0.05 and ^##^ *p* < 0.01 versus the non-irradiated control. * *p* < 0.05, ** *p* < 0.01, and *** *p* < 0.001 versus the UVB-irradiated control.

TGF-β is the major activator of collagen synthesis in human skin fibroblasts. The TGF-β type 1 receptor initiates the synthesis of type 1 procollagen [39]. UV irradiation impairs TGF-β and Smad2/3 signaling, decreasing type 1 procollagen synthesis, and leads to the loss of collagen in the dermis [11]. Smad7 down-regulates TGF-β/Smad signaling through a mechanism of feedback inhibition and reduces the stability of the TGF-β receptor, as well as the activation of Smad2/3 [40,41]. Kim et al. showed that UVB irradiation downregulates the levels of TGF-β and Smad2/3, but that red raspberry reversed this effect [37]. Similarly, in our study, UVB irradiation also reduced the expression of TGF-β and Smad2/3, whereas USM treatment resulted in the upregulation of TGF-β and Smad2/3 (Figure 6). In contrast, Smad7 expression was upregulated following UVB irradiation. However, USM treatment resulted in the downregulation of Smad7 by 32.2, 53.1, and 83.1%, respectively. Therefore, we suggest that USM may protect against UVB-induced collagen degradation through the regulation of TGF-β/Smad signaling.

## 4. Conclusions

In conclusion, the present study showed that USM from PSM attenuated UVB-induced skin damages. USM is a rich source of phytochemicals such as tocopherols, phytosterol, and policosanols, which may contribute to the protective effect of USM against UVB-induced damage in Hs68 cells. The major bioactive compounds among tocopherols, policosanols, and phytosterols were γ-tcopherol, octacosanol (C28), and β-sitosterol, respectively. We found that USM significantly reduced UVB-induced ROS production and attenuated UVB-induced MMP1 and MMP3 mRNA expression by suppressing phosphorylation of MAPKs and AP-1. UVB exposure led to the breakdown of collagen. However, USM resulted in a dose-dependent restoration of the collagen synthesis through stimulation of TGF-β/Smad2/3 signaling. Collectively, the results suggest that USM may be a useful material for use as a functional cosmetic product.

## Figures and Tables

**Table 1:** Phytochemical contents of unsaponifiable matter (USM).

Phytochemicals	Isomers						Total (mg/100 g of USM)
Vitamin E ^a^	α-T 21.66 ± 1.30	β-T – ^c^		γ-T 330.67 ± 4.19		δ-T 10.264 ± 0.47	362.59 ± 5.97
Policosanol ^b^	C24 857.72 ± 41.40	C26 252.88 ± 2.06		C28 1802.98 ± 26.77		C30 847.77 ± 4.19	3761.36 ± 70.29
Phytosterol	Campesterol 3865.16 ± 32.84		Stigmasterol 979.39 ± 2.57		β-Sitosterol 23,016.25 ± 309.87		27,860.80 ± 345.28
Squalene	1028.15 ± 28.68						1028.15 ± 28.68

^a^ Tocopherol (T), ^b^ Tetracosanol (C24), Hexacosanol (C26), Octacosanol (C28), Triacontanol (C30), ^c^ Not detected.
